# Changes in white oak (*Quercus alba*) phytochemistry in response to periodical cicadas: Before, during, and after an emergence

**DOI:** 10.1002/ece3.8839

**Published:** 2022-04-25

**Authors:** Cynthia Perkovich, David Ward

**Affiliations:** ^1^ 4229 Department of Biological Sciences Kent State University Kent Ohio USA

**Keywords:** nitrogen, non‐structural carbohydrate, plant defense relaxation, polyphenols, tannins

## Abstract

Periodical cicadas have mass emergences once every 13 or 17 years. Plants may need to upregulate defense production in response to an emergence. Defense production is energetically expensive, so plants may downregulate their production after periodical cicada populations dissipate. We examined the defensive responses in leaves, branches, and roots of a common host, white oak (*Quercus alba*), prior to, during, and after a 17‐year periodical cicada (*Magicicada* spp.) emergence in western Pennsylvania, United States. During the emergence, total tannins and condensed tannins increased in foliar tissue, while simultaneously decreasing in root tissue compared to the prior and subsequent years. Non‐structural carbohydrates were low prior to the mass emergence but were re‐allocated to belowground storage during the emergence year and dropped thereafter. In the year after the emergence, there was a relaxation of foliar defenses, and root defenses returned to pre‐emergence concentrations. We also tested for differences in damaged and undamaged branches on the same tree during (2019) and the year after the emergence (2020). Both damaged and undamaged branches had significantly greater chemical defenses (polyphenols, total tannins, and condensed tannins) during the emergence than in the following year when there was no emergence. We propose that re‐allocation of resources may help maximize oak tree fitness by moving resources away from areas that are not in immediate threat to areas that are under immediate threat. Changes in aboveground and belowground phytochemistry in response to periodical cicada mass emergences may help us better understand which resource re‐allocation strategies are used by plants to minimize the effects of insect emergencies.

## Graphical abstract

In this study, we take a before, during, and after approach to understand how plants are allocating resources during emergence of periodical cicadas. During these emergences, plant defenses are increased in foliar tissues where the adults are feeding and mating. However, after the emergence, once the nymphs have begun feeding on root tissues, plant defenses are increased in the roots.

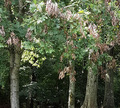

## 1 INTRODUCTION

Plant–insect interactions are subjected to continuous variation, creating a dynamic system. These types of interactions drive the distribution and abundance of plant and insect populations (Maron & Crone, 2006; Moreira et al., 2018; Viskorpi et al., 2019), affect energy and nutrient flow in an ecosystem (Chomel et al., 2016; Yang & Gratton, 2014), and drive evolutionary mechanisms affecting biodiversity within a system (Benton et al., 2021; Moreira et al., 2018; Perkovich & Ward, 2022). The intimate associations between plants and insects in a shared ecosystem can produce beneficial activities, including pollination (Ebling et al., 2018; Losapio et al., 2021) and insect defense of plants (Ashra & Nair, 2022). However, some interactions are harmful, such as intense insect herbivory (Lemoine & Budny, 2022).

To reduce insect attack, plants may use defense strategies such as plant secondary metabolite (PSM) production (Erb, 2018; Karban & Baldwin, 1997; Perkovich & Ward, 2022) or differential resource allocation (Perkovich & Ward, 2021a, 2021b,2021a, 2021b; Wiley et al., 2017). Optimal defense theory predicts that plants increase defenses to minimize immediate herbivore threats (Herms & Mattson, 1992; Stamp, 2003). However, these inducible defenses are energetically costly, which is why they are not consistently produced (i.e., constitutive) (Rhoades, 1979; Stamp, 2003). Furthermore, the growth differentiation balance hypothesis states that physiological costs of defense production limit metabolic processes so that when defenses are produced, resources are allocated away from growth and primary metabolic functions (Fiorucci, 2020; Herms & Mattson, 1992). Resource limitations within an environment create a trade‐off so that plants should allocate resources to defense production to minimize injury without jeopardizing overall fitness (Endara & Coley, 2011; Hattas et al., 2017; Scogings, 2018).

### 1.1 Periodical cicadas

Periodical cicadas (*Magicicada* spp.) emerge every 13 or 17 years (Karban, 2014) in mass emergences that occur in “broods” scattered across the northeastern United States (Dybas & Davis, 1962; Marshall & Cooley, 2000; Williams & Simon, 1995). During emergence years, periodical cicada densities can reach nearly 400 cicadas m^−2^, making their emergences the largest recorded of any insect (Dybas & Davis, 1962; Karban, 2014).

During the developmental years (13 or 17 years), periodical cicada nymphs feed on roots of deciduous trees (Dybas & Lloyd, 1974; Karban, 2014; White, 1980; Williams & Simon, 1995). During emergence years, adults move aboveground and form large chorusing centers for mating in host plants. Host plants of chorusing centers are damaged by the adult cicadas that feed on xylem fluids of stems and foliar tissues (Brown & Chippendale, 1973; Williams & Simon, 1995). After mating, females leave the chorusing center's host plant and move to nearby plants for oviposition. Females cause significant physical injury from “flagging” behavior where the female cicada repeatedly inserts their ovipositors into branch tissues (Karban, 1981; Kritsky, 2004; Williams & Simon, 1995). Flagging behavior continues until the female finds a place to oviposit. The oviposited egg sac blocks all vascular tissues in the branch, often resulting in the subsequent loss of the distal part of the branch (Kritsky, 2004; Williams & Simon, 1995). The density of periodical cicada populations results in numerous oviposition sites on a single tree with mass dieback of branches, occurring in a relatively short period (Kritsky, 2004).

Despite evidence that host plants may respond to periodical cicada damage (Karban, 1983; White, 1981), researchers have largely overlooked these interactions because there is limited evidence that the xylem‐feeding adults are affected by PSM which are generally transported in the phloem (Christensen & Fogel, 2011; Sevanto, 2019). However, host plants often respond to ubiquitous elicitors, such as polysaccharides commonly found in all insects' oral secretions (Jeeter et al., 2004; Matthus et al., 2019). The mechanical damage from periodical cicada feeding and oviposition is sufficient to allow leakage of these polysaccharides into plant cell walls, activating a general defense response and PSM production (Arimura, 2021; Matthus et al., 2019). Even though general responses and PSM production may not influence the behaviors of adult periodical cicadas, PSM production may draw energy and resources away from other plant functions (Douma et al., 2017; Zangerl et al., 1997). The divergence of resources to defense production may leave plants more susceptible to other diseases as well as reducing growth and plant fitness (Clay et al., 2009a; Ostry & Anderson, 1983). We have limited knowledge of the physiological and biochemical responses of plants to periodical cicada emergences (Boyce et al., 2019; Cook & Holt, 2002; Nguyen et al., 2020). Plant responses to large insect emergences, such as the periodical cicadas, offer a unique and untapped resource for further exploration of plant–insect interactions.

### 1.2 Oaks (*Quercus*) as a model study system

Oaks (*Quercus* spp.) are a dominant tree genus spanning the periodical cicada's range and are preferred host trees (Clay et al., 2009b; C. Perkovich & D. Ward, unpublished data). In response to herbivory, oak species have several strategies to prevent further tissue damage (Perkovich & Ward, 2022). One strategy is to increase defensive chemical production (e.g., polyphenols) (Pearse & Hipp, 2012; Wold & Marquis, 1997). Tannins, a class of polyphenols, are organic compounds that precipitate proteins, making the proteins unusable to herbivores (Bernays et al., 1989; Tayal et al., 2020) or may act as toxins in some cases (Barbehenn & Constabel, 2011; War et al., 2018). Tannins are a ubiquitous PSM found in many plant genera and are often thought of as a generalized defense response (Clay et al., 2009a; Salminen & Karonen, 2011; War et al., 2018). They are broken down into two classes—condensed and hydrolysable—with immense structural variability (Clay et al., 2009b; Dixon et al., 2005; Kardel et al., 2013). Even though tannins are a ubiquitous class of PSM, there is often a high degree of variability of tannin concentrations, even within organs of the same plant species and individual (Salminen & Karonen, 2011). A second strategy used by oaks, not mutually exclusive from the defense hypothesis, is to re‐allocate nutrients. For example, oaks have been shown to decrease nitrogen content in the leaves (Frost & Hunter, 2008; Wold & Marquis, 1997) and to re‐allocate non‐structural carbohydrates to belowground storage (Perkovich & Ward, 2021a; Wiley et al., 2017) in response to aboveground tissue removal.

We have limited knowledge of the physiological and biochemical responses of oaks to periodical cicada emergences (Boyce et al., 2019; Cook & Holt, 2002; Nguyen et al., 2020). Plant responses to large insect emergences such as the periodical cicadas offer a unique and untapped resource for further exploration of plant–insect interactions. Using a primarily oak‐dominated forest, we investigated the changes in oak foliar and root chemistry in response to a 17‐year periodical cicada emergence. We used a before, during, and after approach (Quinn & Keough, 2002) to analyze tree phytochemistry before the emergence (when nymphs were feeding belowground), during the emergence (when nymphs were no longer feeding belowground and adults were feeding and ovipositing on aboveground), and after (when adult damage had dissipated, and the next generation was feeding belowground) an emergence. We asked how foliar and root PSM (i.e., polyphenol, tannin, and condensed tannin) and nutritive resources (nitrogen and non‐structural carbohydrates) changed before, during, and after the emergence as the periodical cicadas feeding moved above‐ and belowground. We also asked if foliar upregulation of PSM was only observed in damaged branches.

After analyzing foliar and root phytochemistry the year before an emergence, we made the following predictions:

1. During the emergence, oak trees will express elevated PSM in foliar tissues as a general response to adult periodical cicada feeding and oviposition damage.
2. The energetic expense of PSM production should cause a decrease in foliar nutritive resources (i.e., nitrogen and non‐structural carbohydrates) (Frost & Hunter, 2008; Wold & Marquis, 1997) due to trade‐offs between growth (nutritive resources available for growth) and differentiation (PSM production).
3. Branches damaged by adult periodical cicadas should increase PSM production compared to undamaged branches on the same individual (Tuomi et al., 1988). More specifically, a damaged branch should have an increased concentration of defense compounds compared with undamaged branches, and that same damaged branch will have a relaxation (decline) of these defense concentrations the following year.
4. Concomitantly, non‐structural carbohydrates should be re‐allocated to the roots because of aboveground damage by adult periodical cicadas (Perkovich & Ward, 2021a; Wiley et al., 2017).
5. As a result of decreased herbivory on belowground tissues when periodical cicadas emerge, PSM should decrease belowground during the emergence.
6. Finally, because of diminished need and high cost of PSM production, PSM and nutritive resources should return to pre‐emergence concentrations in respective foliar and root tissues (Huntzinger et al., 2004; Young & Okello, 1998).

## 2 METHODS

### 2.1 Study site and cicada brood VIII

We sampled 50 white oak (*Quercus alba*) trees in Keystone State Park, Westmoreland County, Pennsylvania, United States. Keystone State Park is part of the mixed mesophytic Appalachian Forest with robust oak stands (McCarthy et al., 2001). Brood VIII of the periodical cicada (*Magicicada* spp.) emerges in this forest every 17 years and emerged in late May/early June of 2019 (Cooley et al., 2009; Simon, 1988).

### 2.2 Sampling for foliar changes before, during, and after a cicada emergence

We sampled the 50 white oaks from the bottom of the canopy of forest trees. Because this system is a dense forested area, leaves from the bottom of the canopy were shaded. Collections were taken from the same tree each year on June 10, 2018 (i.e., 1 year before the emergence), June 9, 2019 (i.e., during the emergence), and June 6, 2020 (i.e., 1 year after the emergence). We used growing degree days (GDD_50_) to evaluate climatic changes that could have an effect on oak phytochemistry (Dantec et al., 2014). According to the National Weather Service, there were no significant changes in the number GDD_50_ from the beginning of the year until each sampling date (GDD_50_ range 18.4–20.3, mean = 19.9, data retrieved from National Oceanic & Atmospheric Administration, 2022). During sampling each year, we collected samples of leaf tissues by randomly removing five mature leaves (controlling for ontogenetic changes) from each tree (i.e., 5 leaves from 50 different trees, for a total of 250 leaves sampled). Leaves were placed in a Zip‐loc^®^ bag and placed in a cooler with dry ice. Samples were transported to the lab on the day of sampling and immediately placed in drying ovens. Trees were marked in 2018; sequential years' samplings were collected from the same tree.

Samples were dried at 60°C for 48 h. Oven drying methods are shown to generate losses of total polyphenols compared to flash‐freezing methods (Julkunen‐Tiitto & Sorsa, 2001; Julkunen‐Tiitto & Tahvanainen, 1989; Orians, 1995). However, we sought to compare phenolic compound concentrations among samples, and therefore used the oven‐drying method and standardized concentrations with equivalents (explained below) (Hagerman, 1988, 2011; Mullen et al., 2002; Waterman & Mole, 1994). Once dried, samples from each tree were homogenized and ground using a Wiley mill (mesh size = 2 mm). We extracted polyphenols and tannins using a 70% acetone solution (Graca & Barlocher, 2005; Hagerman, 2011). There are no unique standards for polyphenols or tannins; they are therefore expressed as *equivalents* of the standard used (Hagerman, 2011). Total polyphenols were analyzed using the Prussian Blue assay (Price & Butler, 1977), modified for use on a microplate reader (Hagerman, 2011), and standardized using gallic acid. Total tannins were measured using the radial diffusion assay and standardized against tannic acid (Hagerman, 1987, 2011). Condensed tannins were analyzed using the acid butanol assay for proanthocyanidins (Gessner & Steiner, 2005; Hagerman, 2011) and standardized against quebracho tannin. Non‐structural carbohydrates were extracted using 80% ethanol for sugar and a 1% sulfuric acid solution for starches (see Tomlinson et al., 2013). Total non‐structural carbohydrates were measured using Fournier's (2001) method, using phenol‐sulfuric acid as a solvent to dissolve sugars and starches (see Tomlinson et al., 2013) and standardized in glucose equivalents. A rapid N Exceed^®^ analyzer by Elementar was used to measure percent nitrogen.

### 2.3 Sampling for root chemical changes before, during, and after a cicada emergence

Root samples were collected from the same trees used in the foliar sampling and handled in the same manner as leaves (see above). Root samples were taken after foliar tissues were collected. We collected root samples from roots that were 6 cm in length and between 2.5 and 3.5 mm in diameter to standardize samples between multiple trees. Roots of this diameter are generally used in root storage and not involved in nutrient uptake making this selection optimal for PSM and non‐structural carbohydrate analyses (McCormack et al., 2014). Chemical analyses were conducted as explained above.

### 2.4 Sampling for changes between damaged vs. undamaged branches within individual trees

We performed a paired sampling technique to collect 50 damaged and 50 undamaged branches on 50 white oaks (i.e., one damaged and one undamaged branch from each tree). For this method, we selected 50 different trees (separate from those sampled in the foliar and root analyses) during the emergence (year 2019). For each tree, we analyzed branches from the lower portions of the canopy at a similar height for each tree. From each tree, we collected all leaves from a 30 cm portion of branch tip that had clear flagging damage and classified this as the “damaged” sample for that tree. We then searched the same tree for another branch of similar diameter that did not have flagging damage, collected all leaves from a 30 cm portion of branch tip, and classified this as the “undamaged” branch for that tree. Because periodical cicada oviposition often leads to complete senescence of foliage, we avoided branches with flagging damage whose leaves had already senesced. The sampled branches were marked in 2019 (i.e., during the emergence) and the same branches were re‐sampled in 2020. It is possible that the second year's foliage may have been affected by our sampling in the first year. However, we treated each sample in the same way so that damage was consistent across all samples. Chemical analyses were conducted as previously explained.

### 2.5 Statistical analysis

To analyze tree responses to the periodical cicada emergence, we used a multivariate analysis of covariance (MANCOVA) to minimize Type I statistical error from analyzing multiple dependent variables. Our model included diameter at breast height (DBH) as a covariate to standardize for variation in tree sizes, year (2018, 2019, or 2020) as the independent variables, and total polyphenols, total tannins, condensed tannins, non‐structural carbohydrates, and % nitrogen for both foliar and root tissues (separately) as dependent variables. We then used a univariate analysis of variance (ANOVA), followed by Scheffe's *post hoc* tests for variables that were statistically significant in the MANCOVA.

To analyze phytochemical variability within an individual tree, we again used a MANCOVA analysis with branch diameter in the center of flagging damage as a covariate to standardize for variation in branch sizes among trees, year (2019 or 2020), and damaged or undamaged as independent variables, and foliar total polyphenols, total tannins, condensed tannins, non‐structural carbohydrates, and % nitrogen as dependent variables. We proceeded with an ANOVA and Scheffe's *post hoc* tests for significant dependent variables. All statistical analyses were run using the *vegan* package (Oksanen et al., 2020) in R statistical software (R Core Team, 2021). Graphs were made using *ggplot2* package (Wickham, 2016) in R.

## 3 RESULTS

Oak trees showed significant chemical changes between years (MANCOVA: *Wilk’s λ* = 0.008, *F*_(28, 262)_ = 93.18, *p* < .001). The size of the tree (indexed as diameter at breast height) did not have a significant effect (MANCOVA: *Wilk’s λ* = 0.892, *F*_(14, 130)_ = 1.127, *p* = .34).

### 3.1 Foliar changes before, during, and after a cicada emergence

**FIGURE 1:**
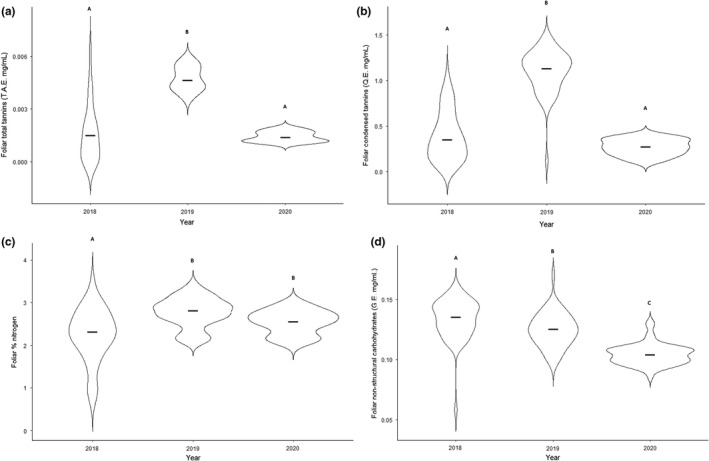
Violin plots of foliar phytochemistry of oak trees before, during, and after a periodical cicada emergence (2019). For each year, individual points represent one oak tree (*n* = 50), and error bars represent 1 standard deviation. (a) Total tannins (measured in tannic acid equivalents (T.A.E. mg/ml) using the radial diffusion assay) and (b) condensed tannins (measured in quebracho equivalents (Q.E. mg/ml) using the acid butanol assay) both significantly increased during the emergence (2019). (c) Foliar nitrogen also significantly increased during the emergence (2019) but did not return to pre‐emergence concentrations in the following year (2020). (d) Foliar non‐structural carbohydrates (measured in glucose equivalents [G.E. mg/ml]) significantly decreased during the emergence (2019) and decreased further the following year (2020). Central horizontal bars represent medians. Dissimilar letters designate significant differences (*p* < .05) among years

The mean foliar tannin and condensed tannin concentrations increased nearly threefold during the emergence year (2019) (ANOVA: *F*_(2, 146)_ = 156.73, *p* < .001 and *F*_(2, 146)_ = 217.98, *p* < .001, respectively, Figure 1a and b). Total tannins and condensed tannins in the year after the emergence (2020) returned to concentrations that were not statistically different from the year preceding the emergence (2018) (Scheffe's *post hoc*: *p* = .37 and *p* = .09, respectively; Figure 1a and b). Foliar nitrogen exhibited a similar significant trend (ANOVA: *F*_(2, 146)_ = 16.83, *p* < .001, Figure 1c), but the year after the emergence (2020) did not return to the concentrations of the year preceding the emergence (2018). Foliar non‐structural carbohydrates decreased during the emergence (2019) and further decreased the following year (2020) (ANOVA: *F*_(2, 146)_ = 36.90, *p* > .001, Figure 1d).

### 3.2 Root chemical changes before, during, and after a cicada emergence

**FIGURE 2:**
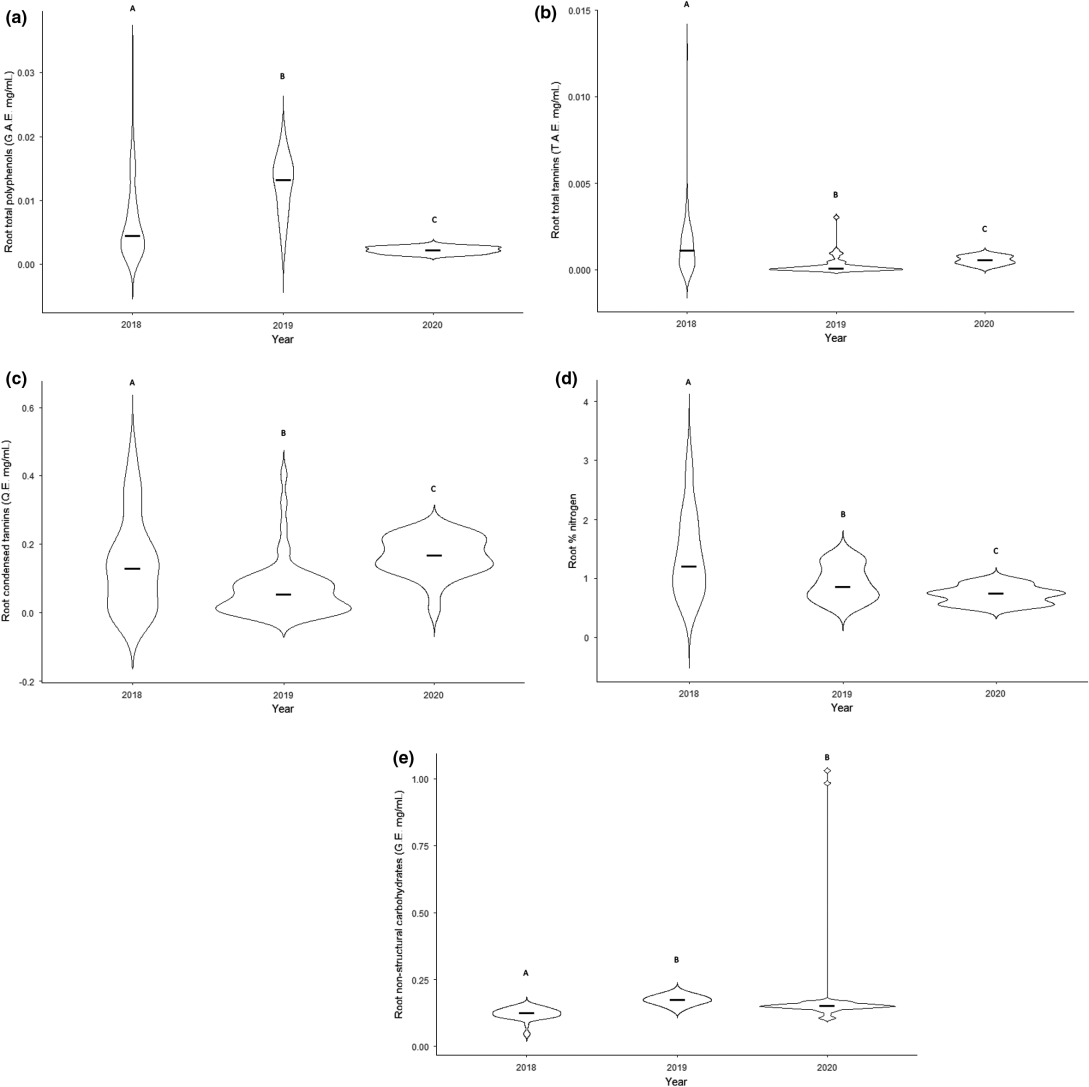
Violin plots of root phytochemistry of oak trees before, during, and after a periodical cicada emergence (2019). For each year, individual points represent one oak tree (*n* = 50), and error bars represent 1 standard deviation. (a) Total polyphenols (measured in gallic acid equivalents (G.A.E. mg/ml) using the Prussian Blue assay) increased during the emergence. (b) Total tannins (measured in tannic acid equivalents [T.A.E. mg/ml]) and (c) condensed tannins (measured in quebracho equivalents [Q.E. mg/ml]) both significantly decreased during the emergence (2019). (d) Foliar nitrogen significantly decreased during the emergence (2019) and remained below pre‐emergence levels the following year (2020). (e) Foliar non‐structural carbohydrates (measured in glucose equivalents [G.E. mg/ml]) significantly increased during the emergence (2019). Central horizontal bars represent medians. Dissimilar letters designate significant differences (*p* < .05) among years

Root polyphenols significantly increased during the emergence (2019) and significantly decreased post‐emergence (2020) (ANOVA: *F*_(2, 146)_ = 63.80, *p* < .001, Figure 2a). Total tannins and condensed tannins followed the same trend, but in the opposite direction of foliar tannins and condensed tannins. These concentrations significantly decreased during the emergence (2019), and significantly increased post‐emergence (2020) (ANOVA: *F*_(2, 146)_ = 14.84, *p* < .001 and *F*_(2, 146)_ = 14.11, *p* < .001, respectively, Figure 2b,c). Root nitrogen significantly decreased during the emergence (2019) and significantly decreased post‐emergence (2020) (ANOVA: *F*_(2, 146)_ = 30.86, *p* < .001, Figure 2d).

### 3.3 Changes in damaged vs. undamaged branches within individual trees

**FIGURE 3:**
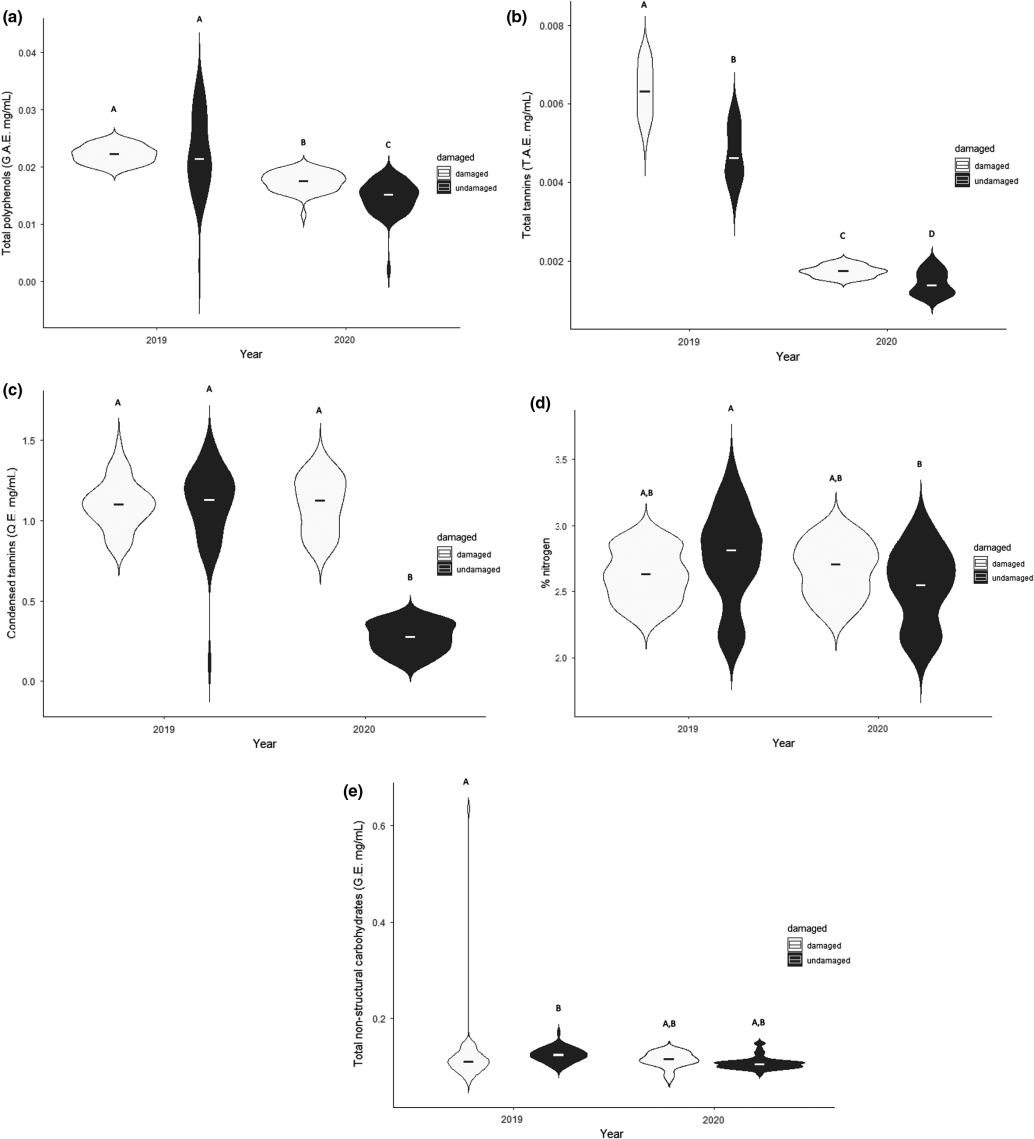
Violin plots showing the phytochemical changes in oak foliage in response to oviposition damage by female periodical cicadas. Damaged branches contained female *flagging* damage, and undamaged were branches without signs of *flagging*. (a) Total polyphenols (measured as gallic acid equivalents [G.A.E. mg/ml]) were the same regardless of damage during the emergence (2019) but significantly decreased post‐emergence (2020). (b) Total tannins (measured as tannic acid equivalents [T.A.E. mg/ml]) in damaged branches were significantly greater than undamaged branches during the emergence (2019) and the following year (2020). (c) Condensed tannins (measured as quebracho equivalents [Q.E. mg/ml]) were the same in damaged and undamaged branches during the emergence (2019), but significantly decreased the following year (2020). Undamaged branches had a significantly greater (d) nitrogen and (e) non‐structural carbohydrates (measured as glucose equivalents [G.E. mg/ml]) during the emergence (2019) than the following year (2020). Central horizontal bars represent medians. Dissimilar letters designate significant differences (*p* < .05) between damaged and undamaged branches within a specific year

There was a significantly greater concentration of defensive compounds (polyphenols, tannins, and condensed tannins) during the emergence (2019) than the year following the emergence (2020) (MANCOVA: *Wilk’s λ* = 0.292, *F*_(5, 192)_ = 190.00, *p* < .001, Figure 3a–c). Post‐emergence (2020), defensive compounds were significantly greater in branches that had been damaged during the emergence (MANCOVA: *Wilk’s λ* = 0.365, *F*_(15, 530)_ = 190.00, *p* < .001, Figure 3a–c).

Damaged branches had significantly lower nitrogen content post‐emergence (2020) than during the emergence (2019) (Scheffe’s *post hoc test*: *p* < .001, Figure 3d). Likewise, non‐structural carbohydrates in damaged branches were significantly lower post‐emergence (Scheffe’s *post hoc test*: *p* = .03, Figure 3e).

## 4 DISCUSSION

During the periodical cicada emergence, oak trees increase chemical defenses and significantly alter nutrient allocation strategies. Costs of defense production may take the form of allocation costs that divert resources away from plant growth and reproduction (Fiorucci, 2020; Herms & Mattson, 1992; Karban & Baldwin, 1997). Induction of defenses is less effective and less cost‐efficient if the plant is unable to reduce investment in expensive defenses when there is no immediate herbivore threat (Huntzinger et al., 2004; Levins, 1968; Young & Okello, 1998). In this study, we found that the foliar increases in defenses and changes in nutrient re‐allocation patterns often relaxed the following year. Contrastingly, the root chemical defenses decreased during the emergence (2019) and returned to the higher, pre‐emergence concentrations the year after the emergence (2020). We hypothesize that the relaxation of root total polyphenols and condensed tannins may be a response to the nymphs discontinuing belowground feeding when they emerge as adults. This relaxation of root defenses may provide necessary allocation of resources to produce foliar defenses as the adult periodical cicadas begin mating. This pre‐supposes that it is the periodical cicada emergence that is imposing much of the induction of plant defenses (Cook & Holt, 2002; Karban, 1982) and not inherent levels of herbivory by other insects that may occupy these trees.

Assuming there is no other significant form of herbivory on these trees, the relaxation of defense chemicals in the roots during the emergence suggests that oak trees are re‐allocating resources to optimize their fitness. Pre‐emergence, most damage is belowground when the nymphs are feeding. However, during the emergence, damage occurs aboveground. In response to changes to location of herbivore attack, plants may alter their defensive phenotype to better protect themselves from the immediate threat and minimize energetic costs (Adler & Karban, 1994). According to Adler and Karban's (1994) *moving targets* model, variation in herbivore threat favors the optimal inducible defense strategy. The optimal inducible defense strategy states that when herbivores are absent, plants will be less defended, investing more in growth. In the presence of a single herbivore population, plants will switch to a more defended state at the cost of reduced growth rate (Adler & Karban, 1994). These predictions are consistent with our data. Periodical cicadas are a single population that feed belowground for 17 years. Concordant with predictions from Adler and Karban's (1994) model, the root total tannins and condensed tannins in our study decreased when the nymphs emerged. Aboveground, the adults stimulated an inducible response, with total tannins and condensed tannins increasing. Oak trees may be altering resource allocation strategies to optimize defenses (Adler & Karban, 1994; Perkovich & Ward, 2021a, 2021b,2021a, 2021b). Acquisition and allocation of resources are plastic traits within a population (Metcalf, 2016; Noordwijk & de Jong, 1986). Individuals with access to fewer resources may use alternative allocation strategies to maximize defense (Metcalf, 2016; van Noordwijk & de Jong, 1986; Ward & Young, 2002). Similarly, individuals experiencing higher levels of stress may also alter allocation strategies to maximize defense (Adler & Karban, 1994; McCormick et al., 2019). Again, assuming that belowground herbivory from non‐periodical cicadas is low during a periodical cicada emergence, individual trees that experience higher levels of damage aboveground may re‐allocate resources away from belowground defense production. The re‐allocated resources may provide additional support to minimize the aboveground threat. Additionally, a study by Cook and Holt (2002) found that oviposition damage was ineffective, or trees were able to sufficiently compensate. We propose that the changes in defense strategy and nutrient re‐allocation may be an evolutionary mechanism allowing for physiological compensation, assuming *ceteris paribus* that the periodical cicadas are causing the maximal negative effect relative to other sources of herbivory or damage.

### 4.1 Foliar responses to periodical cicada emergences

We found total tannin production to be the most common induced response as damaged branches had significantly greater total tannins, but not total polyphenols or condensed tannins during the emergence. Total tannins are a constituent of total polyphenols. However, the increase in total tannin was probably due to ellagitannins or similar hydrolysable tannins because we used tannic acid as the standard (=equivalence) for total tannin analysis. The total polyphenol assay (Prussian Blue) would not necessarily include these changes because a different standard was used. Therefore, it is possible that there were changes in the other antinutritive constituents, but they were undetected due to the methods used. In oaks, tannins are one of the main compounds that present an antiherbivory defense function and have been described to act as an effective deterrent on herbivores (Barbehenn & Constabel, 2011; Bernays et al., 1989; Clausen et al., 1992; Perkovich & Ward, 2020). However, insects, unlike vertebrates, suffer few antinutritional effects of tannins and mainly suffer from toxic effects (Barbehenn & Constabel, 2011; Farahat et al., 2018; Hafeez et al., 2019). We cannot confirm that white oaks were increasing tannin production in response to general injury, such as piercing damage from feeding or if the increased tannin production was in direct response to salivary enzymes of periodical cicadas. Certain biosynthetic pathways (such as jasmonic acid and ethylene pathways) are often activated in response to plant defense elicitors present in insect saliva, frass, or oviposition fluids (Acevedo et al., 2019; Hogenhout & Bos, 2011; Musser et al., 2005). A repertoire of defenses may be synthesized in a specific manner, dependent on the activation cue (Erb et al., 2012). For example, the saliva of the fall armyworm (*Spodoptera frugiperda*) contains phytohormones that elicit a variety of responses in different plant species (Acevedo et al., 2019). This repertoire of defenses is also consistent with Adler and Karban's (1994) *moving targets* model where investments in defense strategies may change to maximize plant fitness.

In the case of many insect herbivores, larvae feed on the same tree where they hatched. The preference performance hypothesis (a.k.a. the “mother‐ knows‐best hypothesis” or the “naïve adaptationist hypothesis”) predicts that insect herbivores should not lay eggs on plants that are heavily fed upon (Courtney & Kibota, 1990; Jaenike, 1978; Valladares & Lawton, 1991). An adaptive strategy is for phytophagous insects to lay eggs on plants that are not heavily consumed (Clark et al., 2011; Lambert et al., 2018; Mayhew, 1997). In the case of non‐feeding adults, we would predict that the induction of defenses would have a negative effect on the root‐feeding nymphs. Although we focused on the effects of the female flagging behavior, it is also possible that they are selecting the trees based on the inclusive fitness to their offspring (Birch, 2017; Hamilton, 1964). That is, by choosing these particular host trees, they are maximizing fitness for the next generation (and thereby improving their own fitness). This is not mutually exclusive from the results we have discussed above.

During the emergence, oak trees displayed a systemic response with similar total polyphenols and condensed tannins in damaged and undamaged branches. There was a differentiation of defensive compounds between damaged and undamaged branches the following year (2020). During the year post‐emergence (2020), total polyphenols, total tannins, and condensed tannins were higher in branches that received flagging damage during the emergence than in undamaged branches.

### 4.2 Non‐structural carbohydrate re‐allocation

As we hypothesized, there was an increase in root non‐structural carbohydrates during the emergence year. Oaks have been shown to increase root storage of non‐structural carbohydrates in response to foliar damage (Perkovich & Ward, 2021a; Wiley et al., 2017). If non‐structural carbohydrates are being shuttled to root storage in response to foliar damage, periodical cicada nymphs may take advantage of this. Nutritional differences in the xylem influence nymphal growth (White & Lloyd, 1985) and excess nutrient availability may stimulate neonatal growth, as shown in other insect species (e.g., Cohen, 2015; Woods et al., 2019). Faster growth provides a competitive advantage for the nymphs that generally compete for resources such as space and nutrients (Karban, 1981; Lloyd & Dybas, 1966; White & Lloyd, 1975).

Female cicadas may select oviposition locations based on whether a plant has low defenses or greater non‐structural carbohydrate concentrations to increase inclusive fitness (Birch, 2017; Hamilton, 1964). There may be selection for periodical cicada nymphs that fall belowground to feed on a tree that has a greater concentration of non‐structural carbohydrates in root storage. Insects are known to develop faster with increased carbohydrate consumption (Kılcı & Altun, 2020; Shen et al., 2017; Silverman, 1995).

### 4.3 Future directions

For this study, we mainly focused on nutritional and antinutritional changes in oak phytochemistry in response to periodical cicada emergences. As stated previously, despite the energy that oaks are investing in response to the periodical cicadas, scientists do not fully understand the effects of these changes on periodical cicada behavior (Simon et al., 2022). Because periodical cicadas only feed on xylem, there may be other chemical signals that play a larger role in host‐seeking behavior, such as volatile organic compounds (VOCs). Oaks are known to produce VOCs in response to injury (Faiola & Taipale, 2020; Volf et al., 2021). VOCs may affect insect host‐seeking behavior by acting as attractants or repellants (Peterson et al., 2018; Smith & Beck, 2013). Injuries due to female flagging behavior may induce VOC emissions that act as a signal for avoidance or preferential ovipositing behaviors (Simon et al., 2022). Furthermore, climate change also causes changes to oak phytochemistry that may negatively affect periodical cicadas (Kye et al., 2021; Moriyama & Numata, 2019). Due to their long development time, many changes may take place aboveground, making the environment they emerge into very different from the environment they were hatched in. The unique life cycle and intimate relationship with forest tree hosts create a unique opportunity for scientists to further explore plant–insect interactions in ecological as well as evolutionary context.

## 5 CONCLUSIONS

Oak tree phytochemistry significantly changes in response to periodical cicada emergences. Changes in production of chemical defense and nutrient re‐allocations are dependent on the plant tissue (i.e., foliar vs. root). In many cases, these changes relax or return to pre‐emergence concentrations the following year. The changes followed by relaxation further support that the trees are directly responding to the periodical cicada emergence and not to long‐term effects of herbivory by other organisms. This is likely an effect of the emergence phenomenon in periodical cicadas. Changes in chemical defense and nutrient concentrations may be an evolutionary mechanism that allows oaks to maximize their fitness during a period of high stress. Re‐allocating resources to better defend tissue that face immediate threats, as predicted by optimal defense theories (Barto & Cipollini, 2005; Rhoades, 1979), is clearly shown by the changes in total tannins during the year of an emergence. Periodical cicada emergences not only induce changes in the concentration of plant defenses but also stimulate a cascade of other phytochemical responses, including the induction of non‐structural carbohydrate storage in the roots.

## CONFLICT OF INTEREST

We have no conflict of interest to disclose.

## AUTHOR CONTRIBUTIONS

**Cynthia Perkovich:**Conceptualization (equal); Investigation (lead); Methodology (lead); Writing – original draft (lead). **David Ward:** Conceptualization (equal); Investigation (supporting); Methodology (supporting); Writing – review & editing (lead).

## Data Availability

Data for this study are archived with Dryad (https://doi.org/10.5061/dryad.ngf1vhhwg).
